# Nitrification Rates Are Affected by Biogenic Nitrate and Volatile Organic Compounds in Agricultural Soils

**DOI:** 10.3389/fmicb.2019.00772

**Published:** 2019-05-14

**Authors:** Santosh Ranjan Mohanty, Mounish Nagarjuna, Rakesh Parmar, Usha Ahirwar, Ashok Patra, Garima Dubey, Bharati Kollah

**Affiliations:** ICAR Indian Institute of Soil Science, Bhopal, India

**Keywords:** nitrification, biogenic nitrate, redox metabolism, mVOCs, 16S rRNA, *amoA*

## Abstract

The processes regulating nitrification in soils are not entirely understood. Here we provide evidence that nitrification rates in soil may be affected by complexed nitrate molecules and microbial volatile organic compounds (mVOCs) produced during nitrification. Experiments were carried out to elucidate the overall nature of mVOCs and biogenic nitrates produced by nitrifiers, and their effects on nitrification and redox metabolism. Soils were incubated at three levels of biogenic nitrate. Soils containing biogenic nitrate were compared with soils containing inorganic fertilizer nitrate (KNO_3_) in terms of redox metabolism potential. Repeated NH_4_–N addition increased nitrification rates (mM NO_3_^1-^ produced g^-1^ soil d^-1^) from 0.49 to 0.65. Soils with higher nitrification rates stimulated (*p* < 0.01) abundances of 16S rRNA genes by about eight times, *amoA* genes of nitrifying bacteria by about 25 times, and *amoA* genes of nitrifying archaea by about 15 times. Soils with biogenic nitrate and KNO_3_ were incubated under anoxic conditions to undergo anaerobic respiration. The maximum rates of different redox metabolisms (mM electron acceptors reduced g^-1^ soil d^-1^) in soil containing biogenic nitrate followed as: NO_3_^1-^ reduction 4.01 ± 0.22, Fe^3+^ reduction 5.37 ± 0.12, SO_4_^2-^ reduction 9.56 ± 0.16, and CH_4_ production (μg g^-1^ soil) 0.46 ± 0.05. Biogenic nitrate inhibited denitrificaton 1.4 times more strongly compared to mineral KNO_3_. Raman spectra indicated that aliphatic hydrocarbons increased in soil during nitrification, and these compounds probably bind to NO_3_ to form biogenic nitrate. The mVOCs produced by nitrifiers enhanced (*p* < 0.05) nitrification rates and abundances of nitrifying bacteria. Experiments suggest that biogenic nitrate and mVOCs affect nitrification and redox metabolism in soil.

## Introduction

Nitrification is a key biogeochemical process for the global nitrogen cycle (Nelson et al., 2016). Therefore, in-depth knowledge on nitrification is essential for agricultural, environmental, and economic reasons. Nitrification of ammonia to nitrate is a two-step process usually performed by two distinct groups of chemolitho-autotrophic microbes (Alfreider et al., 2017), one step oxidizes NH_4_^+^ to NO_2_^1-^, while the other oxidizes NO_2_^1-^ to NO_3_^1-^ (Li Y. et al., 2018). In the first step, most of the NH_4_^+^ is converted to NO_2_^1-^, but a small portion of the N is emitted as N_2_O (Liimatainen et al., 2018). This is produced as a byproduct when the intermediate HNO is produced during the oxidation of NH_2_OH to NO_2_^1-^. HNO is further oxidized to NO_2_^1-^ and finally to NO_3_^1-^ (Weber et al., 2015). Complete ammonia oxidation (comammox) is energetically feasible and bacteria (*Nitrospira* sp.) capable of performing both steps have been identified (Daims et al., 2015). These bacteria encode all enzymes necessary for ammonia oxidation via nitrite to nitrate in their genomes (van Kessel et al., 2015).

Most ammonia oxidizing bacteria (AOB) belong to the *Betaproteobacteria* (β-AOB) (Pan et al., 2018). There are two distinct phylogenetic clusters within the β-AOB, the *Nitrosomonas* cluster and the *Nitrosospira* cluster (Zhao et al., 2015). The *Nitrosomonas* cluster comprises members of the genus *Nitrosomonas*. The *Nitrosospira* cluster comprises the genera *Nitrosospira*, *Nitrosolobus*, and *Nitrosovibrio*. Nitrite (NO_2_^1-^) oxidizing bacteria have been described in four genera; *Nitrobacter*, *Nitrococcus*, *Nitrospina*, and *Nitrospira* (Han et al., 2017). Our understanding of the nitrogen cycle has been revised in the past few years by the discovery of ammonia oxidizing archaea (AOA) (Leininger et al., 2006). AOA are members of the proposed archaeal phylum *Thaumarchaea* (Gribaldo et al., 2010). However, AOA are difficult to cultivate, so some aspects of their physiology and contribution to biogeochemical pathways are still speculative. AOA are found in almost all environments. Crenarchaeotal 16S rRNA gene sequences have been recovered from different environments including Pacific and Atlantic oceans (Flood et al., 2015), lake sediments (Lliros et al., 2014), the guts of animals (Radax et al., 2012), agricultural soils (Tourna et al., 2011), and forest soils (Isobe et al., 2012). Typically AOA greatly outnumber AOB. In soil samples, the copy number of crenarchaeotal *amoA* is one to three orders of magnitude higher than bacterial *amoA* (Wuchter et al., 2006).

Nitrification is carried out by the microbial membrane-bound enzymes. The ammonia monooxygenase (AMO) is responsible for the conversion of NH_3_ to hydroxylamine (Bock and Wagner, 2013). The end product of nitrification, NO_3_^1-^, may binds to cationic molecules present in soil or extracellular microbial molecules. Thus, the NO_3_^1-^ produced by nitrifiers can be different in nature than inorganic NO_3_^1-^. The nitrates produced from nitrification may bind to extracellular complex organic compounds to form “biogenic nitrate.” Contrastingly, inorganic forms of NO_3_ (NaNO_3_, KNO_3_, NH_4_NO_3_, etc.) are in the form of salts. The bonding between NO_3_^1-^ and cations (Na, K, NH_4_, etc) in inorganic NO_3_ fertilizer is stronger than the bonding between NO_3_^1-^ and cellular organic cations in biogenic nitrate. Therefore, nitrate in the inorganic nitrate fertilizer preferably does not bind to cellular organic cations unlike the nitrate produced through nitrification. It is also reported that nitrifiers produce soluble microbial products (SMPs) which serve as supplementary organic substrates for heterotrophic bacteria (Dolinšek et al., 2013). The SMPs are mainly constituted of proteins and humics (Li J. et al., 2018). There is a possibility that after nitrification the product (NO_3_^1-^) binds to SMPs forming “biogenic nitrate.” Like inorganic nitrate, the biogenic nitrate has two main biological functions. Either it is assimilated by plants and microbes (under aerobic condition) (Rubio-Asensio et al., 2014) or it is denitrified when anoxic conditions prevail. Nitrate reduction or denitrification is carried out by dissimilatory nitrate reducing bacteria (Castro-Barros et al., 2017). However, due to its complexation with SMPs, the availability and fate of biogenic nitrate can be different from inorganic fertilizer nitrate (KNO_3_).

Like other microorganisms, nitrifiers can produce volatile organic compounds (VOCs). However, information on the VOCs emitted by nitrifiers is scarce. Microbial VOCs (mVOCs) act as signal molecules for different microorganisms (Insam and Seewald, 2010). The mVOCs can modulate activities of the producing species, or of different microbial species. However, it is unclear how the volatiles produced by nitrifiers influence the activity of nitrifiers and denitrifiers. The manuscript aims to define how the NO_3_^1-^ derived from nitrification is different from that in chemical inorganic nitrate fertilizers.

## Materials and Methods

### Soil Sampling and Characterization

Experiments were carried out using soil samples collected during September 2016 from the experimental fields of the Indian Institute of Soil Science, Bhopal, Madhya Pradesh, India (23.30 N, 77.40 E, 485 m above mean sea level). The soil is a heavy clayey Vertisol (typic Haplustert, WRB code VR), characterized by: 5.7 g kg^-1^ organic C, 225 mg kg^-1^ available N, 2.6 mg kg^-1^ available P, and 230 mg kg^-1^ available K. The textural composition of soil was: sand 15.2%, silt 30.3%, clay 54.5%, electrical conductivity (EC) 0.43 dS m^-1^, and pH 7.5. The soil had 863.24 μM NO_3_^1-^, 0.01 μM Fe^2+^, and 101.02 μM SO_4_^2-^. Concentration of these ions was estimated by wet chemical method as given below (chemical analysis). After collection, the soil was hand-processed after breaking the clods and removing roots and stones. Soil was then passed through 2-mm mesh sieve and was used within 2 days of collection.

### Nitrification and Biosynthesis of Biogenic Nitrate

**FIGURE 1:**
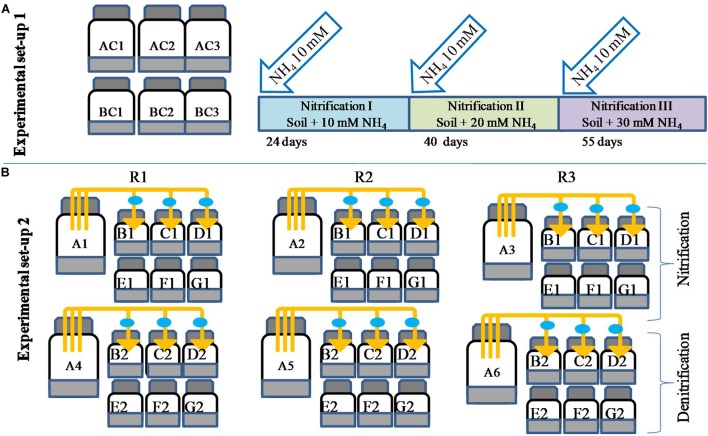
Microcosm design for biosynthesis of biogenic nitrate and estimation of nitrification potential of soil under repeated NH_4_–N amendment (setup 1, **A**). Microcosm setup to evaluate the effect of microbial volatiles (mVOCs) on nitrification and denitrification (setup 2, **B**). Bottles of 250 ml volume contained 50 g soil and were un-amended (AC1–AC3) or amended with 10 mM NH_4_ (BC1–BC3). After complete nitrification of 10 mM NH_4_ (24 days) a second dose of NH_4_ was added (BC bottles) and after complete nitrification (40 days) a third dose of 10 mM NH_4_ was added (BC bottles). The third nitrification stage was completed in 55 days of incubation. The three complete nitrification phases were designated as nitrification I, nitrification II, and nitrification III. The second setup **(B)** was designed to evaluate the effect of mVOCs (A1–A6) on nitrification and denitrification. All experimental treatments included three replicates (R1, R2, R3). The six source bottles (A1–A6) were connected to 18 sink bottles (130 ml volume containing 20 g of soil), shown as B1, C1, D1, B2, C2, and D2. The control bottles (130 ml bottle containing 20 g soil) were without exposure to mVOCs (E1, F1, G1 and E2, F2, G2). The connectors fitted with 0.2-μm filters (filled circle) were used to connect source and sink bottles. After each nitrification stage, one sink bottle and one control bottle were further incubated to determine nitrification and denitrification rates as mentioned the methodology.

Biogenic nitrate is defined here as the nitrate produced via nitrification. To biosynthesize biogenic nitrate, microcosms were prepared where nitrification was carried out three times (Figure 1). The choice of having three repeated NH_4_ additions was based on the fact that in agriculture, N fertilizer is often applied in split doses, and for most crops, three split doses of N are recommended (Arregui and Quemada, 2008). Repeated nitrification resulted in different levels of nitrate (biogenic nitrate). Experiments were carried out in six 1000-ml bottles (Figure 1). Three bottles served as controls (AC1–AC3) and the other three were used for biosynthesis of biogenic nitrate and estimation of nitrification (labeled as BC1–BC3). To each bottle 200 g soil was added and sterilized double distilled water was added to maintain soil at 60% moisture holding capacity (MHC). There was no ammonium amendment to “AC” bottles, while 2 ml of 1 M NH_4_–N (NH_4_Cl) was added the “BC” bottles, giving a final concentration of 10 mM. Soils were mixed thoroughly using a glass rod and bottles were closed with butyl rubber caps. All the bottles were incubated at 30 ± 2°C. At different times, bottles BC1–BC3 were opened and 1-g soil subsamples were taken out to determine NO_3_^1-^ concentrations. Control bottles were also opened for analysis to mimic the treated ones. Nitrate measurement continued till the NO_3_^1-^ concentrations in BC bottles reached a plateau. Nitrification of the first dose of NH_4_–N (10 mM) was referred as “nitrification I.” After nitrification I, 10 mM of NH_4_–N was again added to BC bottles and the same incubation and measurement protocol applied until the NO_3_^1-^ was again stabilized. This second nitrification stage was referred as “nitrification II.” Subsequently, the bottles were again opened and amended with a third dose of 10 mM NH_4_–N in BC bottles. The third nitrification stage was referred as “nitrification III.” The three nitrification stages (nitrification I, II, and III) produced three levels of biogenic nitrate. After completion of each nitrification stage, 20-g soil was taken from the bottles (AC and BC) and incubated to evaluate redox metabolism as described below.

### The Effect of Biogenic Nitrate on Redox Metabolism

To evaluate the effect of biogenic nitrate on redox metabolism, experiments were carried out as described above (AC1–AC3 and BC1–BC3). In addition, 18 130-ml vials were also used for this analysis. Nine vials were kept for evaluating redox metabolism using soil mixed with equivalent amount of inorganic fertilizer nitrate (KNO_3_) as observed in nitrification vials (labeled as A). Another nine vials were used for evaluating redox metabolism using the soil in which biogenic nitrate was produced (labeled as B). Each set of nine vials was represented as three nitrification phases and three replicates. Soil (20 g) from AC1–AC3 and BC1–BC3 bottles (collected after nitrification I, II, and III) were placed into 130-ml serum vials. Soils were mixed with 10 mM of CH_3_COONa, and 50 ml of sterile distilled water. Acetate served as carbon source for anaerobic microbial metabolism. After mixing the contents, bottles were closed with rubber septa and sealed using aluminum crimp seals. Bottles were incubated at 30 ± 2°C with shaking at 100 revolutions per minute (rpm) on an orbital shaker for 30 days. To determine the temporal variation in the reduction of the terminal electron acceptors, 3 ml of slurry from each vial was withdrawn using a syringe (Dispovan, India). Before sampling, the syringes were first flushed with pure N_2_ to maintain anoxic conditions. Slurry samples were processed following standard methods to estimate NO_3_^1-^, Fe^3+^, SO_4_^2-^ (see below). Changes in the concentrations of all electron acceptors (NO_3_^1-^, Fe^3+^, SO_4_^2-^) were measured at each sampling time to estimate the rates of redox metabolism. Headspace gas samples of the vials were analyzed via gas chromatography (see below) to quantify CH_4_ production at the end of the incubation period (30 days).

### Effects of N_2_O and Microbial Volatiles on Nitrification

To test the effect of N_2_O on nitrification, experiments were carried out by placing 20 g soil into six 130-ml sterilized serum bottles. Soils were moistened with sterilized double distilled water to maintain 60% MHC and NH_4_–N was added to a final concentration of 10 mM. After mixing the contents, bottles were closed with rubber septa and sealed with aluminum crimp seals. Three bottles were kept as controls and three were injected with pure N_2_O (Inox Pvt. Ltd., Bhopal, India) to a final mixing ratio of 10 ppmv. Control vials were injected with pure helium (99%) instead of N_2_O. All bottles were incubated at 30 ± 2°C for 30 days. At different incubation, periods bottles were opened and 1 g amounts of soil taken to measure NO_3_^1-^. After each NO_3_^1-^ measurement, bottles were re-incubated with 10 ppmv N_2_O.

To evaluate the effect of mVOCs on nitrification and denitrification, an experiment was set up as shown in Figure 1; 50 g amounts of soil were placed into 250-ml bottles, and sterile double distilled water added to maintain soil at 60% MHC. To each bottle, 10 mM NH_4_–N was added. Bottles were closed with rubber stoppers and tightened with screw caps. Three bottles were controls and six “source bottles” were the source of mVOCs originating from nitrification. Another set of 36 “sink bottles” were 130 ml serum bottles each containing 20 g of soil at 60% MHC. The headspace of one source bottle was connected with three sink bottles using silicon tubes (45 cm long × 0.5 cm internal diameter), each fitted with a needle (1.20 mm × 38 mm) at one end and a 0.2 μm syringe filter (25 mm) and needle (1.20 mm × 38 mm) at the other end. The syringe filters were used to restrict any microbial cross contamination between source bottles and sink bottles. The needles of both ends of the silicon tubes were pierced into the rubber caps of source and sink bottles. Gas phases of source and sink bottles were mixed via repeated (10 times) flushing (withdrawing and injecting) of the headspace of the sink bottles using a 50 ml syringe. A total of 18 sink bottles were connected with six source bottles, and another 18 sink bottles were not connected and served as “mVOCs control.” All bottles were kept in an incubator maintained at 30 ± 2°C in the dark. Headspace gas samples of all sink bottles were analyzed for N_2_O. Nitrification was measured only in the bottles labeled as “controls.”

Nitrification of 10 mM NH_4_–N in the source bottles was repeated three times as described earlier. The three nitrification stages were referred to as nitrification I, nitrification II, and nitrification III. At the completion of each nitrification phase, three sink bottles and three control bottles were removed and used for evaluating nitrification and denitrification rates. To measure nitrification in these bottles, 10 mM NH_4_–N was added and the accumulation of NO_3_^1-^ was determined. Denitrification was measured by adding 10 mM NO_3_^1-^ (KNO_3_) and 50 ml of sterile distilled water. Decline in NO_3_^1-^ concentrations was measured to determine denitrification.

### Chemical Analyses

Soil nitrate content was estimated after extraction with CaSO_4_ and reaction by the phenol disulfonic acid method (Jackson, 1958). Reduced Fe^2+^ was determined by extracting slurries with 0.5 N HCl and ferrozine assay (Stookey, 1970). Sulfate (SO_4_^2-^) content was estimated by extracting slurries with Ca(H_2_PO_4_)_2_ and turbidometric analysis (Searle, 1979). The slopes of regression lines relating the changes in NO_3_–N concentrations with the incubation time were used to determine the potential rates of nitrification or denitritrification (nitrification: μg NO_3_^1-^ produced g^-1^ soil d^-1^; denitrification: μg NO_3_^1-^ consumed g^-1^ soil d^-1^) (Schmidt and Belser, 1982). Potential iron (Fe^3+^) reduction rates were estimated from the increase of Fe^2+^ in slurries over time, and potential sulfate reduction rates were determined from declining SO_4_^2-^ concentrations.

Gas samples of 0.1 ml were withdrawn from the headspaces of the vials using a gas-tight syringe. After each sampling, the headspace was replaced with an equivalent amount of high purity (>99%) helium (He) to maintain atmospheric pressure. Gas analysis was carried out using a gas chromatograph (GC 2010, CIC, India) fitted with flame ionization detector (FID) and electron capture detector (ECD). Gas samples were introduced through the port of an on-column injector. The GC was calibrated before and after each set of measurements using different mixtures of gasses (CO_2_ or CH_4_ or N_2_O) in N_2_ (Inox Gas, Bhopal, India) as primary standards. Primary standards were CO_2_ (500, 1000 ppmv), CH_4_ (10 and 100 ppmv), and N_2_O (1 and 10 ppmv).

To quantify CO_2_ and CH_4_, a Porapak Q column (2 m length, internal diameter 3.175 mm, 80/100 mesh, stainless steel column) was used in combination with the FID. The CO_2_ was quantified after its conversion to CH_4_using a attached methanizer module at 350°C. The injector, column, and detector (FID) were maintained at 120, 60, and 330°C, respectively. N_2_O was estimated using a stainless steel column (2 ft; diameter, 1/8 in) filled with chromosorb 101 (60–80 mesh) coupled to the ECD. The oven temp was 30°C, the injector and detector (ECD) temp were 120 and 330°C, respectively.

### Raman Spectroscopic Analysis of Soil

To test the hypothesis that NO_3_^1-^ derived through nitrification is a complex mixture of NO_3_^1-^ and cellular derived bio-molecules, and to reveal any compositional changes of soil due to nitrification, soil samples were analyzed by Raman spectrophotometry (Guizani et al., 2017). Soil samples of un-nitrified control and after third nitrification (nitrification III) were dried at room temperature. The dried soil samples were ground using a mortar and pestle and passed through a 0.1-mm sieve. Samples were scanned in a high-resolution Raman spectrometer (RamanStation^TM^ 400F, Perkin-Elmer^®^, Beaconsfield, Buckingham-shire, United Kingdom) fitted with Czerny-Turner type achromatic spectrograph. The spectral resolution was 0.4 cm^-1^pixel^-1^ at the spectral range of 200–1050 nm and the source of excitation was a 632.8 nm, air cooled He–Ne laser. Nomenclature of wavelengths and the representing functional groups was based on the earlier publications (Socrates, 2004; Colthup, 2012). Data obtained from the instrument were normalized. Wavelengths representing each functional group were considered for analysis. Intensities of the peaks were added and the average of three replicates was calculated.

### DNA Extraction

DNA was extracted from 0.5 g field soil samples using the ultraclean DNA extraction kit (MoBio, United States) according to the manufacturer’s instructions. The DNA concentrations were determined in a biophotometer (Eppendorf, Germany) by measuring absorbance at 260 nm (A260), assuming that 1 A260 unit corresponds to 50 ng of DNA per μl. DNA extraction was further confirmed by electrophoresis on a 1% agarose gel. The extracted DNA was dissolved in 50 μl TE buffer and stored at -20°C until further analysis.

### Real-Time PCR Quantification of Total Bacteria, Ammonia Oxidizing Bacteria, and Ammonia Oxidizing Archaea

Microbial abundance was estimated from two experimental setups: first with soil samples of un-incubated control, nitrification I, II, and III soils, and second with soil samples exposed to microbial volatiles (mVOCs) of nitrification III and un-exposed controls. The microbial groups estimated were total bacteria, AOB, and AOA. Real-time PCR was performed on a Step one plus real-time PCR (ABI, United States). Reaction mixtures contained 2 μl of DNA template, 10 μl of 2X SYBR green master mix (Affymetrix, United States), and 200 nM of each primer (GCC Biotech, New Delhi). The final volume of PCR reaction mixture was made to 20 μl with PCR grade water (MP Bio, United States). Primers targeting bacterial 16S rRNA genes, bacterial *amoA* genes, and archaeal *amoA* gene were used to quantify the respective microbial abundance. The primers (5’–3’) for bacteria were 1F (CCT ACG GGA GGC AGC AG) and 518R (ATT ACC GCG GCT GCT GG) (Baek et al., 2010); nitrifying bacteria 1F (GGG GTT TCT ACT GGT GGT) and amoA 2R1 (CCC CTC TGG AAA GCC TTC TTC) (Okano et al., 2004); nitrifying archaea arc-Amo-F (STA ATG GTC TGG CTT AGA CG; S = G or C); and arc-amoa-R (GCG GCC ATC CAT CTG TAT GT) (Mutlu and Guven, 2011). Thermal cycling was carried out by an initial denaturizing step at 94°C for 4 min, 40 cycles of 94°C for 1 min, the assay-dependent annealing temperature for 30 s, 72°C for 45 s; and a final extension at 72°C for 5 min. The annealing temperature for 16S rRNA genes was 52°C, and for *amoA* genes of bacteria and archaea were 50 and 52°C, respectively. Fluorescence was measured during the elongation step. Data analysis was carried out with Step one plus software (ABI, United States) as described in user’s manual. The cycle at which the fluorescence of target molecule number exceeded the background fluorescence (threshold cycle [*C_T_*]) was determined from dilution series of target amplicons with defined target molecule amounts. *C_T_* was proportional to the logarithm of the target molecule number. The quality of PCR amplification products was determined by melting curve analysis with temperature increase of 0.3°C per cycle. Standard for bacteria prepared by using *Escherichia coli* strain JM 109 (Promega Inc., United States). For preparing standard for amoA genes of nitrifying bacteria and nitrifying archaea, the PCR products of bacterial amoA and archaeal amoA genes were separately cloned to TOPO TA cloning vector (Invitrogen, United States). Constructed plasmids were transformed into competent cells (One Shot Top 10, Invitrogen, United States). Transformed cells (white colonies) were multiplied in LB broth for 24 h at 37°C and their concentration was estimated using a Biophotometer (Eppendorf, Germany). Plasmids from the *E. coli* or transformed cells were extracted using a plasmid extraction kit (Axygen, United States). Concentration of plasmids was quantified and expressed as ng μL^-1^. Serial dilution for each plasmid was prepared and real-time PCR carried out. Standard curve for each gene was prepared by plotting plasmid concentration (representing cell number or gene copies) versus C_T_ values (Supplementary Table S1).

### Statistical Analysis

All statistical analyses were carried out using the “agricolae” packages of the statistical software R (2.15.1) (Ihaka and Gentleman, 1996). Data obtained were presented as arithmetic mean of three replicated observations. Effect of factors (NH_4_ amendment) on the parameters (nitrification, denitrification, Fe^3+^ reduction, SO_4_^2-^ reduction, CH_4_ production, and microbial abundance) was tested by analysis of variance (ANOVA). Low *P*-value and high F statistics indicated significant impact of the factors on the variables. To define the significant difference among the treatments, Tukeys honestly significant difference (HSD) test was performed.

## Results

### Nitrification Activity of Soil

**FIGURE 2:**
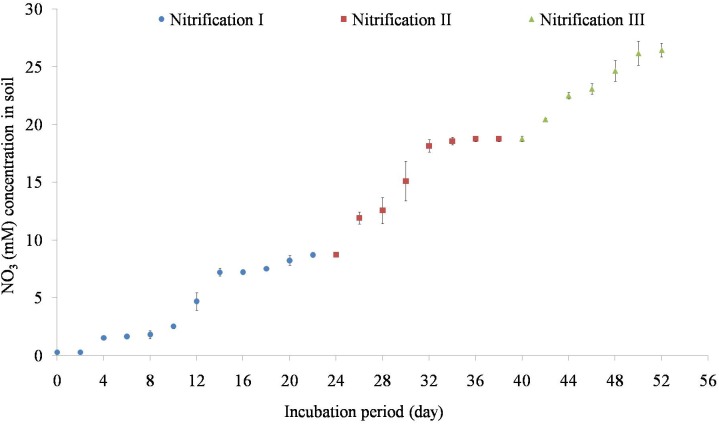
Temporal variation of nitrification in response to 10 mM NH_4_–N amendment. Nitrification was estimated as the increase in NO_3_^1-^ concentration afterNH_4_–N amendment. After complete nitrification, soils were again amended with 10 mM NH_4_ for a second and a third time to complete three nitrification stages (nitrification I, nitrification II, and nitrification III). Each data point represents an arithmetic mean with standard deviation of three replicates.

Variation of NO_3_^1-^ concentrations during repeated stages of nitrification is shown in Figure 2. Nitrification increased steadily after 5 days of incubation. Nitrification of the first dose of 10 mM NH_4_–N occurred within 24 days. Subsequent amendment of NH_4_–N stimulated nitrification. The second dose of 10 mM NH_4_–N was nitrified by 40 days while the third dose of 10 mM NH_4_–N was nitrified by 55 days. The added NH_4_–N was nitrified by about 84% in nitrification I, 92% in nitrification II, and 87% in nitrification III stages. Potential nitrification rates (PNRs) increased with repeated nitrification (Table 1). PNR of fresh soil was 0.49 mMg^-1^ soil d^-1^ while the PNR of nitrification III soil was highest of 0.65 mMg^-1^ soil d^-1^.

**Table 1:** Nitrification and microbial abundance in soil after nitrification of three successive 10 mM NH_4_–N amendments.

Nitrification	Nitrification rate (mM NO_3_^1-^ produced g^-1^ soil d^-1^)	Bacteria (×10^6^ 16S rRNA gene copies g^-1^ soil)	Nitrifying bacteria (× 10^4^ bacterial amoA gene copies g^-1^ soil)	Nitrifying archaea (× 10^4^ archaeal amoA gene copies g^-1^ soil)
Unincubated control		5.00 ± 1.00	4.00 ± 1.46	6.00 ± 1.00
Nitrification I	0.49 ± 0.01	16.67 ± 5.69	32.33 ± 6.43	58.00 ± 8.19
Nitrification II	0.56 ± 0.09	29.00 ± 7.81	66.00 ± 11.53	71.00 ± 16.52
Nitrification III	0.65 ± 034	43.67 ± 4.51	102.33 ± 8.50	94.33 ± 7.77


*The three nitrification stages were referred as nitrification I, nitrification II, and nitrification III. Microbial abundance was estimated after complete >80% oxidation of the added ammonium. Soil without added ammonium served as a control. Values represent arithmetic means and standard deviation of three replicates.*

### Effect of Nitrification on Microbial Abundance

Abundances of total bacteria, nitrifying bacteria, and nitrifying archaea all increased after repeated nitrification (Table 1). The bacterial population varied from 5 to 43.67 (×10^6^ cells g^-1^ soil). The nitrifying bacterial population ranged from 4 to 102 (×10^4^ cells g^-1^ soil) and the nitrifying archaeal population varied from 6 to 94.33 (×10^4^ cells g^-1^ soil). The lowest abundances were measured in control treatments and the highest in the nitrification III soil samples.

### Effect of Nitrification on Redox Metabolism

**FIGURE 3:**
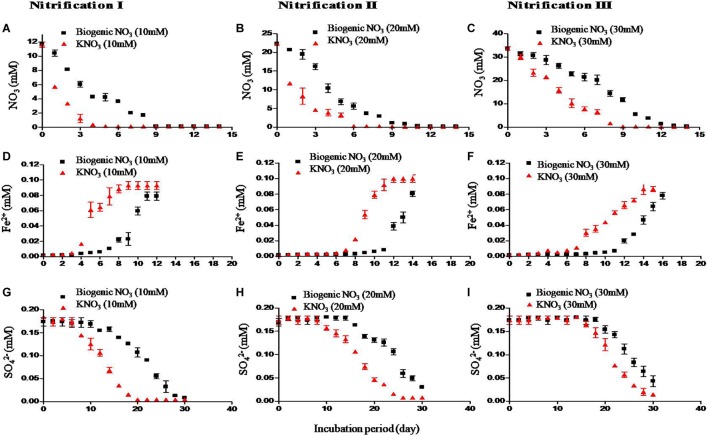
Effect of nitrification on redox metabolism. Soil samples after three nitrification stages were incubated to undergo redox metabolism. Inorganic NO_3_^1-^ (KNO_3_) was used to compare with biogenic NO_3_^1-^ (i.e., nitrate produced from nitrification). First row – denitrification (NO_3_^1-^ reduction) **(A–C)**, second row – iron (Fe^3+^) reduction **(D–F)** measured as increase of Fe^2+^ concentration, and third row – SO_4_^2-^ reduction **(G–I)**. The three nitrifications stages were nitrification I (left), nitrification II (center), and nitrification III (right). Each data point represents an arithmetic mean and standard deviation of three replicates.

Redox metabolism followed the classical trend of sequential reduction of terminal electron acceptors (Figure 3), starting with NO_3_^1-^ reduction followed by Fe^3+^ reduction, SO_4_^2-^ reduction, and CH_4_ production. Soil amended with inorganic KNO_3_ exhibited detectable nitrate reduction after 2 days and complete denitrification within 5 days. Iron reduction peaked at 5 days and SO_4_^2-^ reduction after 2 weeks. Potential redox metabolic rates are presented in Table 2. Denitrification rates increased with NO_3_^1-^ concentration originating from either nitrification phases or KNO_3_. However, the denitrification rate was lower in the soil that had undergone nitrification than compared to the KNO_3_ treated soil. Denitrification may have been inhibited by biogenic nitrate. The reduction rate of Fe^3+^ was also lower in the nitrification soil. Similarly, the reduction of SO_4_^2-^ was also low in the nitrification soil. Production of CH_4_ was estimated after the end of incubation. CH_4_ production was low in nitrification soil compared to non-nitrification soil (Table 2).

**Table 2:** Influence of biogenic NO_3_^1-^ and inorganic fertilizer KNO_3_ on soil denitrification rate, iron reduction rate, sulfate reduction rate, and methane production rate.

Source of NO_3_^1-^	Nitrification phases	Denitrification rate (mM NO_3_^1-^ reduced g^-1^ soil d^-1^)	Iron reduction rate (μM Fe^3+^ reduced g^-1^ soil d^-1^)	Sulfate reduction rate (μM SO_4_^2-^ reduced g^-1^ soil d^-1^)	CH_4_ production (μg CH_4_ produced g^-1^ soil)
Biogenic NO_3_^1-^	Nitrification I	1.22 ± 0.04	5.29 ± 0.26	9.41 ± 0.14	0.54 ± 0.04
	Nitrification II	2.03 ± 0.02	2.95 ± 0.10	9.35 ± 0.03	0.41 ± 0.08
	Nitrification III	2.80 ± 0.04	2.89 ± 0.10	9.19 ± 0.06	0.38 ± 0.04
Inorganic fertilizer NO_3_^1-^	Nitrification I	1.63 ± 0.17	8.55 ± 0.58	10.89 ± 0.17	0.63 ± 0.11
(KNO_3_)	Nitrification II	2.84 ± 0.18	5.84 ± 0.10	10.45 ± 0.19	0.57 ± 0.10
	Nitrification III	4.01 ± 0.22	5.37 ± 0.12	9.56 ± 0.16	0.46 ± 0.05


*The three nitrification stages were referred as nitrification I, nitrification II, and nitrification III. Soil sub-samples collected at the end of the three nitrification phases were incubated for redox metabolism. Values represent arithmetic means and standard deviations of three replicates.*

### Statistical Analyses

Analysis of variance indicated that NH_4_–N addition significantly and positively influenced nitrification (*p* < 0.0001) (Table 3). It also significantly influenced NO_3_^1-^ reduction (*p* < 0.0001), and Fe^3+^ reduction (*p* < 0.01) compared to or inorganic nitrate amendment. However, SO_4_^2-^ reduction and CH_4_ production were not significantly affected. Abundances of 16S rRNA genes, *amoA* genes of nitrifying bacteria, and *amoA* genes of nitrifying archaea were significantly (*p* < 0.0001) influenced by the NH_4_ amendment.

**Table 3:** Analysis of variance (ANOVA) to determine the effect of NH_4_ amendment on nitrification, denitrification, sulfate reduction, CH_4_ production, abundance of bacterial 16S rRNA genes, *amoA* of nitrifying bacteria, and *amoA* of nitrifying archaea.

Parameters	F statistics	*P*-value
Nitrification	80.37	< 0.0001
NO_3_^1-^ reduction	2997	< 0.0001
Fe^3+^ reduction	21	< 0.01
SO_4_^2-^ reduction	1.349	0.285
CH_4_ production	3.955	0.187
16S rRNA genes of eubacteria	52.73	< 0.0001
amoA genes of nitrifying bacteria	103.5	< 0.0001
amoA genes of nitrifying archaea	16.21	< 0.01


### Raman Spectra of Soil in Response to Nitrification

**FIGURE 4:**
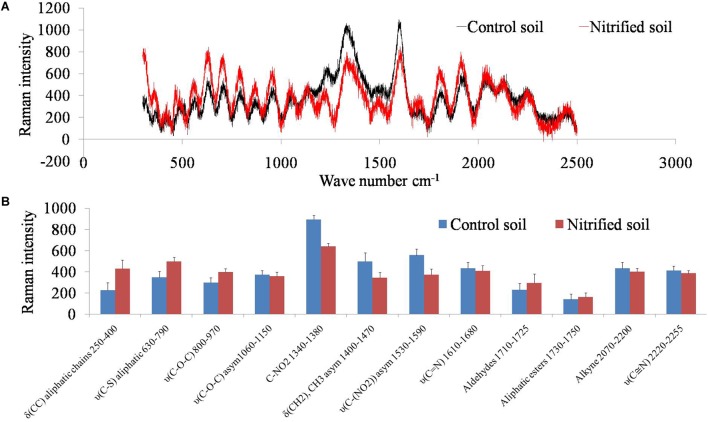
Raman spectra of the non-nitrified (control) and nitrified (after nitrification III) soil **(A)**. The *x*-axis represents wavenumber cm^-1^ and the *y*-axis represents Raman intensity. Raman intensity of different functional groups (wavenumber cm^-1^) of nitrified (nitrification III) and control soils **(B)**. The *x*-axis represents functional groups and the *y*-axis represents Raman intensity. Data points are arithmetic means and standard deviations of three replicates.

Soil samples were scanned by a Raman spectrometer to examine how soil organic carbon changed due to the metabolism of nitrifiers (Figure 4). Nitrified soil (nitrification III) had high absorbance for the wavelengths (wavenumbers cm^-1^) between 500–1000 and 1500–2000. Absorbance intensity was low for the wavelengths ranging from 1200–1600. Raman intensity for the above wavelengths was plotted for both the samples (Figure 4). Nitrification increased the concentration of C–C, C–S, C–O–C molecules and decreased C–NO_2_, and CH_2_ molecules.

### Production of N_2_O and CO_2_ From Soil During Nitrification

Headspace N_2_O and CO_2_ production were measured from control soil (no added nitrogen) and soil after the three nitrification stages (Table 4). N_2_O production rates varied from 4.06 to 19.39 μg g^-1^ soil d^-1^. The lowest rate was in control soil and the highest was in nitrification III soil. The amount of headspace CO_2_ varied from 465 μg g^-1^ soil d^-1^ in control soil to 649 μg g^-1^ soil d^-1^ in nitrification III soil. The values of N_2_O varied significantly among the treatments.

**Table 4:** Production rates of N_2_O, CO_2_, potential rates of nitrification and denitrification in soil in response to repeated ammonium additions.

Nitrification	N_2_O production (μg produced g^-1^ soil d^-1^)	CO_2_ production (μg produced g^-1^ soil d^-1^)	Potential nitrification rate (mM NO_3_^1-^ produced g^-1^ soil d^-1^)	Potential denitrification rate (mM NO_3_^1-^ reduced g^-1^ soil d^-1^)
Control	4.06 ± 0.06	465 ± 50.16	0.42 ± 0.01	1.37 ± 0.05
Nitrification I	11.97 ± 0.84	575 ± 94.85	0.47 ± 0.02	1.38 ± 0.06
Nitrification II	15.87 ± 1.80	605 ± 36.02	0.57 ± 0.01	1.38 ± 0.01
Nitrification III	19.39 ± 2.61	649 ± 39.02	0.84 ± 0.06	1.38 ± 0.04


*The three successive nitrification stages are referred as nitrification I, nitrification II, and nitrification III. Soil without added ammonium served as control. Values represent arithmetic means and standard deviations of three replicates.*

### Effect of N_2_O and Nitrifying Microbial Volatiles on Nitrification and Denitrification

**FIGURE 5:**
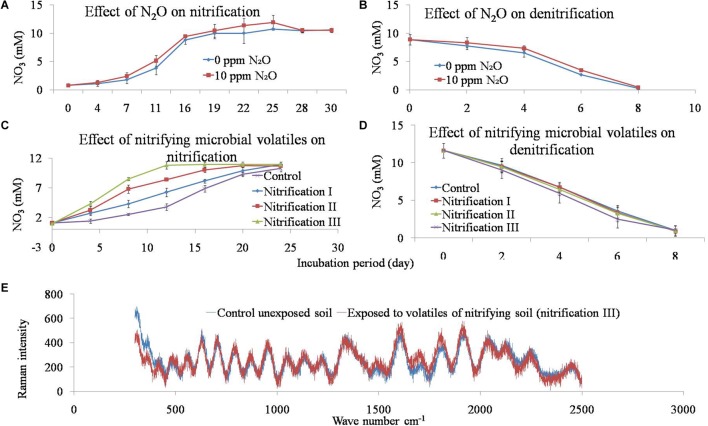
Effect of N_2_O and microbial volatile organic compounds (mVOCs) on nitrification and denitrification activity in soil **(A,B)**. The mixing ratios of N_2_O were either 0 or 10 ppm. To evaluate the effect of microbial volatiles (mVOCs) produced during nitrification on nitrification and denitrification activity, soils were exposed to the gas phase of soils during nitrification I, nitrification II, and nitrification III stages. Soil without exposure served as controls. After exposure, soils were incubated to determine nitrification **(C)** and denitrification **(D)** rates. The *x*-axis represents incubation period and the *y*-axis represents NO_3_^1-^ concentration. Data points are arithmetic means and standard deviations of three replicates. Raman spectra of soil under the influence of nitrifying microbial volatiles **(E)**. Soils were exposed to nitrifying microbial volatiles (nitrification III) or not exposed (control). The *x*-axis represents wavenumber cm^-1^ and the *y*-axis represents Raman intensity. Data points are arithmetic means of three replicates.

The effect of N_2_O on the nitrification and denitrification was evaluated by exposing soil to 0 or 10 ppm of N_2_O. Production of NO_3_^1-^ was measured during nitrification, while the decline of NO_3_^1-^ was measured during denitrification. Nitrification of 10 mM NH_4_ was completed in 3 weeks whereas the denitrificaion of NO_3_^1-^ (∼10 mM) was completed within 8 days. Added N_2_O had no significant effect on nitrification and denitrification (Figure 5).

The effect of volatiles originating from nitrification was tested on nitrification and denitrification (Figure 5). The composition of nitrifier-derived mVOCs was not evaluated in this study because the primary aim was to reveal the influence of mVOCs on nitrification and denitrification. Soils were exposed to microbial volatiles of three repeated nitrification (nitrification I, II, and III) phases. The mVOCs originating from nitrifiers significantly stimulated nitrification (Figure 5). Time required for complete nitrification of the added NH_4_ was significantly reduced due to the volatiles. Nitrification rates (mM NO_3_^1-^ produced g^-1^ soil d^-1^) varied from 0.425 in control soil to 0.844 in nitrification III soil. Nitrification and denitrification values of the controls remained unchanged over the three nitrification phases. However, mVOCs of nitrifiers did not influence denitrification. Potential denitrification rates (mM NO_3_^1-^ reduced g^-1^ soil d^-1^) varied from 1.37 to 1.38 with no statistical difference among the treatments (Table 4).

### Microbial Abundance in Response to Microbial Volatiles

The effect of nitrifying mVOCs on the soil microbial abundance was estimated by quantifying the 16S rRNA genes of eubacteria, *amoA* genes of nitrifying bacteria, and *amoA* genes of nitrifying archaea. Exposure of soils to mVOCs of nitrification (nitrification III) did not increased microbial abundance in soils (Table 5). This indicated that the mVOCs were not a substantial substrate for growth of soil microorganisms. However, prior exposure of soils to mVOCs and subsequent incubation for nitrification resulted in a significant increase in the growth of nitrifying bacteria. Probably, the mVOCs may have activated the nitrifiers in some way resulting high microbial abundance.

**Table 5:** Effect of microbial volatiles (mVOCs) produced during nitrification on the abundance of different microbial groups.

Nitrifying microbial volatiles	Nitrification	Eubacteria (×10^6^ 16S rRNA gene copies g^-1^ soil)	Nitrifying bacteria (×10^4^ bacterial amoA gene copies g^-1^ soil)	Nitrifying archaea (×10^4^ archaeal amoA gene copies g^-1^ soil)
Un-exposed	Before nitrification	5.67 ± 0.57	4.33 ± 0.57	6.49 ± 0.65
	After nitrification	43.67 ± 4.50	103.33 ± 6.11	93.00 ± 8.54
Exposed to nitrifying volatiles (mVOCs)	Before nitrification	6.00 ± 1.00	4.67 ± 1.11	6.67 ± 1.15
	After nitrification	64.67 ± 3.51	195.67 ± 12.85	139.33 ± 16.16


*Soils were exposed to the volatiles originating from nitrification over three successive ammonium additions. Soils without exposure served as an un-exposed control. After exposure to mVOCs, soils were amended with 10 mM NH_4_–N and incubated. Microbial abundance was estimated before and after nitrification of this added ammonium. Values represent arithmetic means and standard deviations of three replicates.*

### Raman Spectra of Soil Exposed to Nitrifying Microbial Volatiles

Soils after exposure to the nitrification III and control (unexposed) treatments were analyzed by Raman spectra (Figure 5). The Raman intensity across the total wavelengths of the two samples was mostly equivalent with no apparent change.

## Discussion

NO_3_^1-^ influences (mostly negatively) reduction of Fe^3+^ (Ionescu et al., 2015), SO_4_^2-^ (Ontiveros-Valencia et al., 2013), and methanogenesis (Rissanen et al., 2017). Denitrification is thermodynamically more favorable than the reduction of other electron acceptors (Fe^3+^, SO_4_^2-^, CO_2_). This influence of inorganic nitrate on redox metabolism is well understood. However, the role of biogenic NO_3_^1-^ on redox metabolism is not known. Therefore, the interaction between nitrification (which produces biogenic nitrate) and redox metabolism was explored.

Soils were amended with 10 mM NH_4_–N and the progress of nitrification was monitored. The PNRs measured were similar to those observed in other soils (Fierer et al., 2001). The nitrification was repeated three times to generate three levels of NO_3_^1-^ (biogenic nitrate). Nitrification rates increased over repeated NH_4_–N amendments, as did the abundance of both nitrifying bacteria and archaea. After each nitrification stage, the soils were evaluated for redox metabolism. Soil samples were incubated under flooded moisture regime to test the effect of the biogenic nitrate versus inorganic nitrate (control) on redox metabolism. Biogenic nitrate inhibited reduction of electron acceptors compared to inorganic NO_3_^1-^. This is reasonable as any compound or processes that inhibited denitrification will ultimately affect the reduction of subsequent terminal electron acceptors (Fe^3+^, SO_4_^2-^, CO_2_).

**FIGURE 6:**
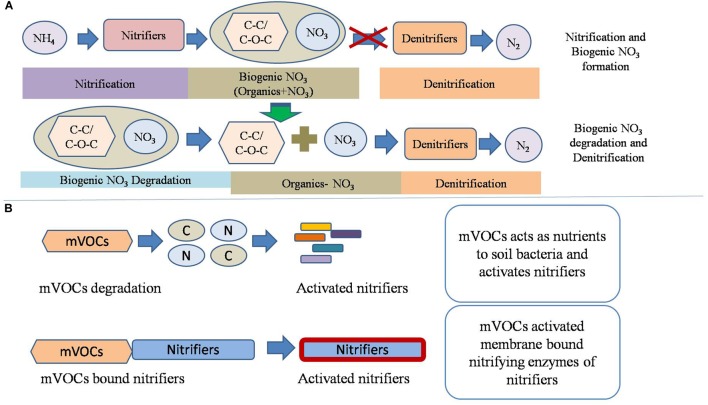
Conceptual model of nitrification and its interaction with the redox metabolism. **(A)** The proposed mechanism of biogenic nitrate formation and of its interaction with denitrification. Biogenic nitrates are produced by the binding of NO_3_^1-^ with extracellular organic compounds, possibly aliphatics. The biogenic nitrates are degraded before onset of redox metabolism under anaerobic conditions. **(B)** The proposed role of microbial volatile organic compounds (mVOCs) emitted by nitrifiers on soil nitrification. It is hypothesized that mVOCs acts as nutrients for proliferation of certain microbial groups and/or bind to cell membrane proteins to activate nitrifiers.

The production of biogenic nitrate via nitrification significantly (*p* < 0.05) inhibited redox metabolism compared to the addition of inorganic NO_3_^1-^. Several soil factors may have been affected by the nitrification phase. One possibility is that nitrifiers produced biomolecules which inhibited the redox metabolism. To identify those biomolecules, soils of non-nitrified control soil and soil from the nitrification III stage were analyzed by Raman spectrometer. Soils of nitrification III were selected for Raman spectra analysis because these soils had undergone maximum nitrification. Raman spectra differentiated soil of control (with an equivalent amount of KNO_3_) from soils of nitrification III. Nitrification increased the abundance of functional groups including C–C, C–S, C–O–C. Spectra also indicated that nitrification decreased the amount of functional groups including C–NO_2_, CH_2_/CH_3_, C–NO_2_, C–N, esters, and alkynes. Therefore, the inhibition of redox metabolism by nitrification may have been due to the presence and/or absence of these functional groups. Under anaerobic conditions, denitrifiers oxidize aliphatic bonds (C–C and C–O–C) to CO_2_ through NO_3_^1-^ dependent oxidation (Zedelius et al., 2011). Theoretically, the occurrence of aliphatics would stimulate the redox metabolism by acting as substrates for the anaerobic microorganisms. However, in the current experiment, they were correlated with reduced redox metabolism. Probably, the biogenic nitrate was less reactive (denitrifying) than the inorganic NO_3_^1-^ as mentioned above. This could be due to the complex interaction or bonding between NO_3_^1-^ and the extracellular aliphatics. In control (non-nitrified soil), the C–NO_2_ functional groups were high. Spectral data indicated occurrence of biogenic nitrate in soil that has undergone nitrification. Biogenic nitrate is a complex form of nitrate containing organic molecules. The organic molecules can be short- or long-chain aliphatics. The complex structure and bonding between aliphatics and NO_2_^1-^ /NO_3_^1-^ makes it less reactive to undergo denitrification (Figure 6). It has been found that organic compounds may inhibit denitrification (Gilbert et al., 1997). Probably, the biogenic NO_3_^1-^ was denitrified after separation of NO_3_^1-^ and aliphatics, which might have been carried out by anaerobic microorganisms (Rabus et al., 2016). The processes of decomposition of the biogenic nitrate by microorganisms probably delayed the availability of NO_3_^1-^ for denitrification. Thus, due to the delayed denitrification, there was delay in the reduction of subsequent electron acceptors comprising Fe^3+^, SO_4_^2-^, and CH_3_COO^1-^ (CH_4_ production).

It was observed that unlike other microbial activities, nitrification progressed steadily in spite of a constant increase in NO_3_^1-^ concentration. Therefore, the formation of biogenic NO_3_^1-^ may be a mechanism used by nitrifiers to block the feedback inhibition of NO_3_^1-^ to nitrification. Production of CO_2_ did not significantly vary with nitrification potential. However, N_2_O production varied significantly among the treatments. Nitrous oxide was generally produced from nitrification, because active nitrification (continuous increase in the NO_3_^1-^ concentration) was observed over the incubation period. N_2_O production through denitrification cannot be ruled out, because some denitrification might have occurred in the soil microaggregates. However, NO_3_^1-^ production from NH_4_ was consistent and there was no decline in the NO_3_^1-^ level. Therefore, the denitrification mediated N_2_O production could be marginal. A follow-up experiment was carried out to determine the effect of N_2_O on nitrification and denitrification. It was observed that there was no significant effect of N_2_O on nitrification and denitrification.

Apart from CO_2_ and N_2_O, other gaseous products emitted by soil microorganisms include mVOCs. Soil microbes produce VOCs including alkenes, alcohols, ketones, terpenes, benenoids, pyrazines, acids, and esters (Lemfack et al., 2013). Microbial volatiles act as signal molecules to other microorganisms, plants, and animals (Insam and Seewald, 2010). The composition of mVOCs originating from nitrification was beyond the scope of this research, which aims only to provide primary information about the influence of mVOCs on nitrification and denitrification. Based on the current study, conceptual models were developed depicting the potential interaction of mVOCs and nitrifiers (Figure 6). This experiment suggested that mVOCs stimulated nitrification, but no effect on denitrification. Probably, the mVOCs acted as signal molecules for the nitrifiers and stimulated their activity (nitrification). Exposure of soil to mVOCs did not increase the abundance of bacteria, nitrifying bacteria, and nitrifying archaea, suggesting that mVOCs stimulated the nitrifiers by increasing cell activity. Many bacteria decompose VOCs in soil (Tyc et al., 2016). The degraded products could have played important role in the activation of microbial population, resulting in high nitrification rates compared to the unexposed control. Soils after exposure to mVOCs were further tested by Raman spectrometer to evaluate if the volatiles altered chemical composition. However, mVOCs did not change the measured soil properties. We propose that the mVOCs stimulate nitrifiers by acting as signal molecules rather than altering the soil properties.

## Conclusion

The current experiment addressed four key questions about nitrification. First, how does nitrification progress under repeated N amendment? Second, how does nitrification influence redox metabolism? Third, how does the nitrate produced from nitrification (biogenic nitrate) differ from inorganic nitrate? Fourth, do the nitrifiers communicate by means of VOCs? Nitrification activity was observed under three repeated N amendments. Nitrification increased steadily in respect to the NH_4_–N amendment, due to increasing abundance of nitrifying bacteria and nitrifying archaea. After each nitrification stage, soils were incubated to undergo redox metabolism. An initial nitrification phase inhibited redox rates compared to the addition of an equivalent amount of inorganic NO_3_^1-^ (KNO_3_). Raman spectra of the nitrified soils revealed an increased concentration of aliphatics. Based on these observations, it was hypothesized that during nitrification, biogenic nitrates are produced by complex interaction (bonding) between NO_3_^1-^ and the aliphatics, and that this biogenic nitrate is less reactive toward denitrification than is inorganic nitrate. Nitrifiers emitted VOCs which stimulated nitrification. Nitrification was accelerated by both VOCs and biogenic nitrate. The current experiment mostly indicated the formation of biogenic nitrate and mVOCs by nitrifiers which regulate nitrification and redox metabolism. However, there is need of comprehensive studies on the biochemical characteristics of biogenic nitrate and mVOCs to better understand the nitrification. Further studies are also warranted with other soil types as well as under field conditions to verify complex interaction between biogenic nitrate, VOCs, and nitrification.

## Author Contributions

SRM conceptualized the experiments, and drafted the manuscript. MN executed experiments and performed most of the wet chemical analysis. RP assisted in setting up experiments. UA contributed in analyzing redox moieties of soil samples. GD performed qPCR reactions to quantify functional genes. BK analyzed data statistically and contributed in drafting and revising the manuscript.

## Conflict of Interest Statement

The authors declare that the research was conducted in the absence of any commercial or financial relationships that could be construed as a potential conflict of interest.
