# Supplementary material for: Nitrification Rates Are Affected by Biogenic Nitrate and Volatile Organic Compounds in Agricultural Soils
Source: Front Microbiol. 2019 May 14;10:772. doi: 10.3389/fmicb.2019.00772 (PMC6527594; doi:10.3389/fmicb.2019.00772)
Supplement: Supplementary file 1 [file Table_1.DOCX]

Supplementary Table 1. Standards of real time PCR derived to quantify bacterial 16S rRNA genes, bacterial amoA genes and archaeal amoA genes in soil samples. Concentration of DNA and threshold (Ct) values were Ln transformed to derive linear models. The model for 16S rRNA fitted as y = -0.103x + 3.112 (R^2^=0.923), where y was DNA concentration and x was Ln Ct values. The models for bacterial amoA gene fitted as y = -1.438x + 5.309 (R² = 0.929) and for archaeal amoA gene as y = -2.286x + 5.621 (R² = 0.856), where y was Ln Ct values and x was Ln DNA concentration. Triplicate values were used to predict models.

| Ln DNA concentration(ng/µl) | | | Ln Ct | | |
| --- | --- | --- | --- | --- | --- |
| 16 rRNA | Bacterial amoA | Archael amoA | 16S rRNA | Bacterial amoA | Archael amoA |
| 4.605 | -0.693 | -0.29 | 2.671 | 0.821 | 1.70 |
| 4.605 | -0.693 | -0.29 | 2.672 | 0.781 | 1.74 |
| 4.605 | -0.693 | -0.29 | 2.676 | 0.804 | 1.62 |
| 4.382 | -0.470 | -0.06 | 2.721 | 0.807 | 1.72 |
| 4.382 | -0.470 | -0.07 | 2.695 | 0.761 | 1.84 |
| 4.382 | -0.470 | -0.07 | 2.678 | 0.780 | 1.73 |
| 3.912 | -0.183 | 0.22 | 2.712 | 0.747 | 1.57 |
| 3.912 | -0.183 | 0.22 | 2.721 | 0.735 | 1.70 |
| 3.912 | -0.183 | 0.22 | 2.719 | 0.755 | 1.57 |
| 3.689 | 0.223 | 0.63 | 2.695 | 0.680 | 1.42 |
| 3.689 | 0.223 | 0.63 | 2.712 | 0.653 | 1.45 |
| 3.689 | 0.223 | 0.63 | 2.710 | 0.695 | 1.45 |
| 2.996 | 0.916 |  | 2.767 | 0.615 |  |
| 2.996 | 0.916 |  | 2.772 | 0.615 |  |
| 2.996 | 0.916 |  | 2.753 | 0.615 |  |
| 2.303 |  |  | 2.811 |  |  |
| 2.303 |  |  | 2.778 |  |  |
| 2.303 |  |  | 2.827 |  |  |
| 0.000 |  |  | 3.177 |  |  |
| 0.000 |  |  | 3.157 |  |  |
| 0.000 |  |  | 3.153 |  |  |
